# Retraction: Implementations of Virtual Reality for Anxiety-Related Disorders: Systematic Review

**DOI:** 10.2196/110891

**Published:** 2026-09-11

**Authors:** JMIR Publications Editorial Office

**Affiliations:** 1 JMIR Publications Toronto, ON

The JMIR Publications Editorial Office is retracting the article “Implementations of Virtual Reality for Anxiety-Related Disorders: Systematic Review” [1] due to concerns regarding the integrity of the peer review process.

Author Julie Prescott responded but did not specifically agree or disagree with the decision to retract the article. Author Theodore Oing did not respond to communications.
